# Inclusive climate perception and voice behavior among new generation employees in China: the role of workplace friendship and relational attribution

**DOI:** 10.3389/fpsyg.2026.1795956

**Published:** 2026-04-15

**Authors:** Lulu Zhang, Ling Ling, Jincen Xiao

**Affiliations:** 1 School of Economics and Management, Chongqing University of Posts and Telecommunications, Chongqing, China; 2 School of Public Administration, Guizhou University, Guiyang, China; 3 School of Management, Xihua University, Chengdu, China

**Keywords:** attribution theory, broaden-and-build theory of positive emotions, inclusive climate perception, relational attribution, voice behavior, workplace friendship

## Abstract

**Introduction:**

In today’s competitive business environment, employee voice, intended to improve organizational effectiveness, is critically important for sustainable development. This study aimed to investigate the relationship between inclusive climate perception and voice behavior among new generation employees in China. It also examined the mediating role of workplace friendship and explored the moderating effect of relational attribution. A moderated mediation model was constructed.

**Methods:**

This study collected data using a multi-wave and multi-source research design. A total of 259 new generation employees and their line managers’ paired data were used in the following empirical analysis.

**Results:**

The results showed that inclusive climate perception positively affected voice behavior among China’s new generation employees, and workplace friendship played a mediating role in this relationship. Relational attribution moderated the mediating effect of workplace friendship.

**Conclusion:**

Drawing on the broaden-and-build theory of positive emotions and attribution theory, this study expands the antecedents of voice behavior and highlights the roles of workplace friendship and relational attribution in the relationship between inclusive climate perception and voice behavior among new generation employees in China.

## 1 Introduction

Employee voice is critical for organizational effectiveness, innovation, and learning (Morrison, 2014). Employee voice is an important form of challenging organizational citizen behavior (Morrison, 2011), and it is also viewed as an image-risk behavior in the workplace (Weiss and Morrison, 2019). Voice behavior refers to employees offering suggestions, comments, or ideas to improve their team, department, or organization, with the primary goal of improvement rather than criticism (LePine and van Dyne, 2001), and it is related to a broad spectrum of antecedents (Chamberlin et al., 2017), such as perceptions of organizational justice (Zhang et al., 2014), felt responsibility (Chamberlin et al., 2017), positive workplace climate (Lee et al., 2014), and inclusive climate (Paolillo et al., 2021). Extensive studies have clarified the intrinsic mechanisms between inclusive leadership and voice behavior (Guo et al., 2022; Jiang et al., 2022).

In the context of China, Zhen and Tang (2026) found that, compared to previous generations, newer generation employees—referred to as the post-reform generation, were born after the 1980s (“Post-80s”) (Xu et al., 2023)—have gradually become the backbone of the workforce (Ren et al., 2018). New generation employees tend to be goal- and achievement-oriented (Wong et al., 2008), have a higher desire for value, respect, and recognition (Weyland, 2011), and exhibit greater trust in more inclusive leaders and lower trust in less inclusive ones (Zhen and Tang, 2026). Paolillo et al. (2021) mentioned that little research has focused on inclusive climate and voice behavior. Moreover, few studies have systematically investigated the relationship between inclusive climate perception and voice behavior among new generation employees in China. Existing research emphasizes the psychological mechanisms through which environmental factors, along with individual differences, influence employee voice behavior (Paolillo et al., 2021). Detert and Burris (2007) recommended that future research needs to advance the comprehension of contextual factors leading to voice behavior within these psychological mechanisms. Therefore, it remains unclear what the intrinsic mechanisms and boundary conditions are in this relationship.

To deepen our understanding of the mechanisms underlying the relationship between inclusive climate perception and voice behavior among China’s new generation employees, this study proposes that workplace friendship mediates the relationship between inclusive climate and voice behavior, and that relational attribution moderates the relationship between inclusive climate and workplace friendship. Lee et al. (2014) suggested that scholars need to unpack the “black box” between a participative climate (i.e., inclusive climate) and employee voice behavior. In response to this suggestion, Paolillo et al. (2021) examined the mediating effect of basic psychological needs satisfaction between inclusive climate and voice behavior. However, it is not enough to explain its mediating mechanism by relying solely on self-determination theory.

Broaden-and-build theory states that certain discrete positive emotions (e.g., joy, interest, contentment, pride, and love) can broaden people’s momentary thought–action repertoires and build their enduring personal resources, including physical, intellectual, social, and psychological resources (Fredrickson, 2001). Workplace friendship refers to a positive, informal, nonromantic, and voluntary relationship between coworkers (Pillemer and Rothbard, 2018). As an important psychological resource, the mediating role of workplace friendship requires further investigation. Moreover, Guanxi is regarded as a fundamental aspect of the workplace in China (Danford and Zhao, 2012). Some studies indicate that self-centeredness and individualism are generally core features among new generation employees (Xu et al., 2023). The effect of inclusive climate on voice behavior cannot be isolated from new generation employees’ relational attribution; which remains underexplored. Therefore, drawing on the broaden-and-build theory of positive emotions and attribution theory, this study constructs a moderated mediation model to explain the mechanism of inclusive climate perception on voice behavior.

This study aims to make contributions to the literature on inclusive climate and voice behavior in three aspects. First, although prior studies have investigated the influence of inclusive climate on voice behavior from the self-determination perspective (Paolillo et al., 2021), the intrinsic mechanism of this effect is still poorly understood. Therefore, by adopting a positive emotion perspective (e.g., workplace friendship), this study enhances the understanding of voice behavior among China’s new generation employees by proposing a novel underlying mechanism of its effects. Second, the boundary condition of inclusive climate perception and its outcomes is an important yet underexplored issue. We propose an attribution angle and take relational attribution as a vital boundary condition for the effectiveness of an inclusive climate. Third, we integrate the broaden-and-build theory of positive emotions and attribution theory, construct a moderated mediation model, and offer novel theoretical insights by examining how relational attribution moderates the mediating effect of workplace friendship. This study systematically illustrates the positive effect of inclusive climate perception on China’s new generation employees’ voice behavior.

## 2 Literature review and hypotheses development

### 2.1 Inclusive climate perception and voice behavior

Burris (2012) indicated that voice behavior has two dimensions: challenging voice behavior, which focuses on modifying organizational conventions and represents change-oriented voice behavior, and supportive voice behavior, which emphasizes preserving existing organizational policies, practices, or procedures. Employees who speak up in challenging ways, especially when such behavior conflicts with managers’ viewpoints, may lead managers to react unfavorably (Burris, 2012) and receive lower performance appraisals (De Dreu and Weingart, 2003).

As a kind of proactive organizational citizenship behavior, voice behavior plays a vital role in organizational change (LePine and Van Dyne, 1998), innovation (Wei and Zhang, 2010), and sustainable development (Duan et al., 2016). Research has indicated that employees value and believe that generationally diverse teams increase team performance (Becker et al., 2020). In line with the increasing heterogeneity of human resources, and the diverse backgrounds of new generation employees in China, organizations need to cultivate an inclusive climate to enhance voice behavior among new generation employees.

Climate is a classic area of research in organizational psychology, and the relationship between climate and performance is supported (González-Romá et al., 2009). Inclusive climate is conceptualized as comprising three dimensions (Nishii, 2013). First, the fair implementation of employment practices emphasizes the elimination of relational or demographic bias and focuses on equitable human resource practices. Second, the integration of differences aims to create an open interaction pattern in which every employee can express and engage their true self-concept (Kahn, 1990), thereby capturing interpersonal integration among diverse employees (Nishii, 2013). Third, inclusion in decision making encourages new generation employees to participate as insiders in organizational processes, such as information sharing and decision making, thereby enabling them to feel like members of the organization with access to job resources and opportunities to participate in organizational decision-making (Shore et al., 2011).

Inclusive climate can stimulate employees’ psychological safety and organizational identification, thereby increasing their organizational citizenship behavior (Jansen et al., 2017) and voice behavior (Li et al., 2023). Existing research has demonstrated that team environment, such as team processes in learning, play an important role in enabling individual development and improving work outcomes (Gemmano et al., 2026). Therefore, when China’s new generation employees perceive an inclusive climate, they will speak up more. Thus, we propose the following hypothesis:

*Hypothesis 1*: Inclusive climate perception is positively related to China’s new generation employees’ voice behavior.

### 2.2 Mediating role of workplace friendship

Positive emotions and psychological experiences may be important explanatory mechanisms between various antecedent variables and voice behavior (Fu et al., 2012). The broaden-and-build theory of positive emotions elaborates the essential role of positive emotions such as happiness, satisfaction, interest, and love in the individual’s cognitive mindset and social contacts with others (Fredrickson, 2001). Compared with negative emotions, individuals who have positive emotions can broaden their cognition and attention in a short period (Fredrickson, 2013), which makes them more open and flexible and improves their creative problem-solving skills (Fredrickson, 2001).

In addition, positive emotions are important psychological, physical, cognitive, and social resources, which increase individuals’ participation in organizational activities and their interaction and sharing with colleagues (Fredrickson and Branigan, 2005). Previous studies have shown that employees with positive emotions show more voice behavior (Liu S.M. et al., 2021; Zhou et al., 2022).

In the field of positive psychology, workplace friendship refers to a positive, informal, nonromantic, and voluntary relationship that contains communal norms and socioemotional goals characteristics between coworkers in the same organization (Pillemer and Rothbard, 2018). In this study, we assume that inclusive climate perception may influence voice behavior through the mediating role of workplace friendship.

Generally speaking, inclusive climate emphasizes establishing a fair and unbiased organizational climate (Nishii, 2013), thus helping new generation employees experience equitable relationships with their colleagues and promoting them to build workplace friendships. Researchers have found that workplace friendship can promote employees’ knowledge sharing (Cao and Zhang, 2020), and organizational citizenship behavior (Wang et al., 2023). As mentioned before, high-quality workplace friendships can generate positive emotions like happiness, love, and pleasure, which drive new generation employees to speak up and express their views or advice about improving organizational development.

According to the above statement, we propose the following hypothesis:

*Hypothesis 2*: Workplace friendship mediates the relationship between inclusive climate perception and China’s new generation employees’ voice behavior.

### 2.3 Moderating role of relational attribution

Workplace friendship has four features, including informality, voluntariness, communal norms, and socioemotional goals (Pillemer and Rothbard, 2018). It is not only built upon interaction opportunities and similarities with coworkers (Zhang et al., 2021), but also relies on creating a supportive work environment or climate of trust with each other, which strengthens workplace friendship (Zhang et al., 2021).

Inclusive climate emphasizes satisfying employees’ belongingness and uniqueness needs and focuses on both similarities and distinctiveness (Shore et al., 2011), and aims at creating an equal, inclusive, and integrative organizational climate under a workforce diversity background (Nishii, 2013), and it provides core elements for building workplace friendship. However, it is not enough to construct workplace friendship only from the new generation employees’ perception of an inclusive climate, as it also depends on their interpretation of being included.

Relational attribution refers to the explanations made by an individual that assign the cause of a performance-related event to the relationship the individual has with another person (Eberly et al., 2011). Drawing on attribution theory, we contend that relational attribution may weaken the positive relationship between inclusive climate perception and workplace friendship.

In other words, when relational attribution is high, friendship is already high, and employees interpret the reason of being included as the good relationship with the organization and its members; they may adopt a “give and take” social exchange process to attribute the relationship (Liden et al., 2016), and then enhance their desire to build and maintain workplace friendship. However, when relational attribution is low, friendship starts at lower levels, and inclusive climate exerts a stronger positive effect that satisfies new generation employees’ belongingness and uniqueness needs and helps construct deeper workplace friendships. Therefore, we propose that relational attributions negatively moderate the relationship between inclusive climate perception and workplace friendship.

As mentioned before, we contend that China’s new generation employees’ perception of an inclusive climate promotes voice behavior through the mediating effect of workplace friendship. However, we also argue that the magnitude of this indirect effect depends on the employee’s attribution of being included. When employees make relational attributions, they may feel involuntary constraints and exchange norms, which aim to realize some instrumental goals (Pillemer and Rothbard, 2018), thus enhancing the need for workplace friendship, and they may speak up more.

Therefore, through integrating Hypotheses 1, 2, and 3, we hypothesize that relational attribution moderates the mediating effect of workplace friendship, meaning that the mediating role of workplace friendship is more positive between inclusive climate perception and voice behavior when relational attribution is low rather than high.

According to the above statement, we propose the following hypotheses:

*Hypothesis 3*: Relational attribution moderates the positive relationship between inclusive climate perception and workplace friendship, such that the relationship is stronger when relational attribution is low.

*Hypothesis 4*: Relational attribution moderates the mediating effect of workplace friendship on the relationship between inclusive climate perception and voice behavior, such that the mediating effect is stronger when the level of relational attribution is lower.

**Figure 1:**
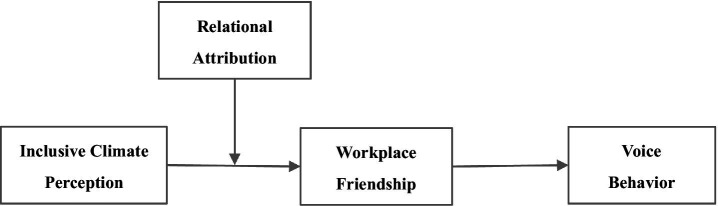
Theoretical model.

We presented the theoretical model in Figure 1.

## 3 Method

### 3.1 Procedures and samples

We conducted a questionnaire survey to collect the data needed for subsequent empirical analysis. Participants were new generation employees from a high-tech company in Chongqing, a city in China, engaged in software development, cloud computing, and artificial intelligence. Inclusion is one of the company’s core cultures, which aims to create an equitable, relaxed, and enjoyable workplace climate, which is suitable for many new generation employees in this company. We emphasized that the survey was voluntary, anonymous, and that the data were only used for academic research.

We collected data in two waves and from two sources to minimize potential common method bias. Employees needed to evaluate inclusive climate perception, relational attribution, and their personal demographic information at time point one. A month later, we conducted the second phase follow-up questionnaire survey; employees who participated in and completed the first survey were required to complete the workplace friendship scale in this study. Meanwhile, the voice behavior scale was rated by employees’ line managers to decrease social desirability bias.

In the first wave survey, we sent 310 questionnaires to employees and obtained 282 completed questionnaires, and the response rate was 90.97%. In the second wave survey, we distributed 282 questionnaires to employees and their line managers separately, and we obtained 273 questionnaires from employees and 264 questionnaires from line managers, with response rates of 96.81 and 93.62%, respectively. We removed missing or mismatched questionnaires, and eventually obtained 259 leader–follower pairwise data. Female participants accounted for 54.40, and 45.60% were male. Among the participants, 91.10% were under 35 years old, and 8.90% were aged between 36 and 39 years. In addition, 95.40% of participants held undergraduate degrees or higher, and 4.60% held college degrees.

### 3.2 Measures

#### 3.2.1 Inclusive climate perception

This scale was developed by Nishii (2013). The scale consists of three subscales with 15 items: equitable employment practices, integration of differences, and inclusion in decision making. The sample items are “This unit has a fair promotion process,” “This unit is characterized by a non-threatening environment in which people can reveal their ‘true’ selves,” and “In this unit, everyone’s ideas for how to do things better are given serious consideration.” The items were rated from 1 (strongly disagree) to 5 (strongly agree). In this study, the Cronbach’s alpha coefficient was 0.78.

#### 3.2.2 Relational attribution

This scale was developed by Eberly et al. (2017). The scale is unidimensional and consists of two items. We designed a scenario event: “Assuming that you are now in a unit with high level of inclusive climate, you perceive the unit climate to be fair in personnel hiring, promotion, and other related personnel decisions, and you experience that interpersonal exchanges among unit members do not differentiate based on status, identity, values, or interests. Meanwhile, you can truly perceive that individual differences are respected and integrated to improve organizational efficiency. In this scenario, why are you included by the work unit and its members?” According to this scenario event, participants needed to evaluate and judge their perception with the following two items: “As a result of your partnership with this unit and its members,” and “Because you have a good relationship with this unit and its members.” The items were rated from 1 (strongly disagree) to 5 (strongly agree). In this study, the Cronbach’s alpha coefficient was 0.70.

#### 3.2.3 Workplace friendship

Workplace friendship was assessed with a nine-item scale developed by Sun and Jiao (2012) in the Chinese organizational context. The sample item is “I have the opportunity to talk informally or make small talk with others in this unit.” The items were rated from 1 (strongly disagree) to 5 (strongly agree). In this study, the Cronbach’s alpha coefficient was 0.73.

#### 3.2.4 Voice behavior

Voice behavior was assessed with a six-item scale from Burris (2012), and the scale was rated by line managers. The sample item is “This employee challenges me to deal with problems around here.” The items were rated from 1 (strongly disagree) to 5 (strongly agree). In this study, the Cronbach’s alpha coefficient was 0.70.

#### 3.2.5 Control variables

Employees’ gender, age, and education level were controlled in this study.

## 4 Results

### 4.1 Descriptive statistics

Table 1 shows the means, standard deviations, and correlations of the variables. As presented in this table, inclusive climate perception is positively related to workplace friendship (*r* = 0.58, *p* < 0.01) and voice behavior (*r* = 0.54, *p* < 0.01). Relational attribution is positively related to workplace friendship (*r* = 0.13, *p* < 0.01).

**Table 1:** Descriptive statistics and correlations between the key variables.

Variable	M	SD	1	2	3	4	5	6	7
1. Gender	1.54	0.50	1						
2. Age	1.69	0.70	−0.16^*^	1					
3. Education	3.10	0.52	−0.03	−0.09	1				
4. ICP	4.37	0.32	0.04	0.13^*^	−0.06	1			
5. RA	4.27	0.77	0.08	−0.01	0.10	0.09	1		
6. WF	4.43	0.36	0.00	0.11	−0.11	0.58**	0.13*	1	
7. VB	4.15	0.49	−0.06	0.19^**^	−0.06	0.54^**^	−0.04	0.58^**^	1

^*^p < 0.05; ^**^p < 0.01 (two-tailed test); *N* = 259. ICP, inclusive climate perception; RA, relational attribution; WF, workplace friendship; VB, voice behavior.

### 4.2 Measurement of models

We used a confirmatory factor analysis to measure the fit of the research model. As shown in Table 2, compared with the other three models, the four-factor model, which is the theoretical model of this study, has the best fit statistics (chi-square to degree of freedom (*χ^2^*/df) = 1.98; the comparative fit index (CFI) = 0.90; non-normed fit index (NNFI) = 0.90; standardized root mean square residual (SRMR) = 0.60; root mean square error of approximation (RMSEA) = 0.06). The results suggest that the variables in this study have good discriminant validity.

**Table 2:** Results of confirmatory factor analysis.

Model	*χ*^2^/df	CFI	NNFI	SRMR	RMSEA
4-factor model	1.98	0.90	0.90	0.06	0.06
3-factor model	2.07	0.89	0.88	0.07	0.06
2-factor model	2.22	0.87	0.86	0.07	0.07
1-factor model	2.40	0.85	0.84	0.075	0.07

*N* = 259. For the 3-factor model, workplace friendship and voice behavior were combined into one factor; for the 2-factor model, inclusive climate perception, workplace friendship, and voice behavior were combined into one factor.

### 4.3 Hypotheses test

We performed hierarchical regression with SPSS 23.0 to test Hypotheses 1 to 3; the results are presented in Table 3. Inclusive climate perception had a positive, direct relationship with voice behavior (*β* = 0.80, *p* < 0.001), and Hypothesis 1 was supported.

**Table 3:** The results of direct effect and indirect effect.

Variables	Workplace friendship			Voice behavior		
	Model 1	Model 2	Model 3	Model 4	Model 5	Model 6
Control variables						
Gender (1 = male)	0.01	−0.02	−0.01	−0.03	−0.06	−0.50
Age	0.05	0.01	0.02	0.12^**^	0.07	0.07^*^
Education	−0.07	−0.06	−0.05	−0.04	−0.02	0.01
Independent variables						
ICP		0.64^***^	0.56^***^		0.80^***^	0.45^***^
Moderator						
Relational attribution		0.04^+^	0.05^*^			
Interaction						
ICP*RA			−0.06^***^			
Mediator						
Workplace friendship						0.55^***^
*R* ^2^	0.02	0.35	0.38	0.04	0.31	0.41
Δ*R*^2^		0.33	0.03		0.27	0.11

*N* = 259; except for total *R*^2^ and Δ*R*^2^ rows, entries are unstandardized regression coefficients; ^+^*p* < 0.10; ^*^p < 0.05; ^**^p < 0.01; ^***^p < 0.001.

The results of the mediation analysis were presented in Model 6. Compared with Model 5 (*β* = 0.80, *p* < 0.001), we found that inclusive climate perception was still positively related to voice behavior (*β* = 0.45, *p* < 0.001), but the regression coefficient decreased. Therefore, workplace friendship had a partial mediating effect between inclusive climate perception and voice behavior. Moreover, we performed a bootstrapping procedure with 5,000 replicated samples, which demonstrated that the indirect effect of workplace friendship was significant, as the 95% confidence interval did not include “0” (indirect effect = 0.35, 95% CI = [0.22, 0.49]), and Hypothesis 2 was supported.

**Figure 2:**
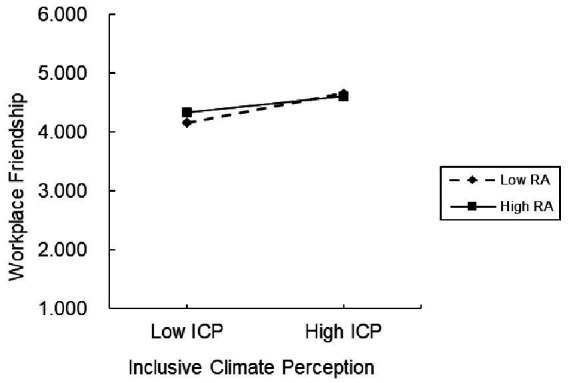
Moderating effect of relational attribution.

The results of the moderation analysis were presented in Model 3. We centered inclusive climate perception, relational attribution, and their interaction term, and then entered these variables into the regression model sequentially. The result indicated that the interaction term of inclusive climate perception and relational attribution was negatively related to workplace friendship (*β* = −0.06, *p* < 0.001). We adopted Aiken and West’s (1991) suggestion, and calculated simple slopes at one standard deviation above and below the mean of relational attribution. We drew the moderating effect diagram as shown in Figure 2, and found that a low level of relational attribution led to a more positive effect of inclusive climate perception on workplace friendship (simple slope = 0.78, *p* < 0.001), compared with a higher level (simple slope = 0.41, *p* < 0.001). Therefore, Hypothesis 3 was supported.

Table 4 presents the results of the moderated mediation analysis. We used the bootstrapping process macro procedure developed by Preacher et al. (2007) with 5,000 replications to analyze the difference in the conditional indirect effects under low and high levels of relational attribution. We found that the mediating role of workplace friendship between inclusive climate perception and voice behavior is stronger and significant at the low level (effect size = 0.42, SE = 0.09, CI = [0.24, 0.61]) but weaker and still significant at the high level (effect size = 0.22, SE = 0.07, CI = [0.10, 0.40]), with a significantly different estimate (difference effect = −0.20, SE = 0.10, CI = [−0.48, −0.07]). Furthermore, the index of the moderated mediation effect of relational attribution is significant (index = −0.13, SE = 0.07, CI = [−0.32, −0.04]). Therefore, Hypothesis 4 was supported.

**Table 4:** The results of the moderated mediation effect.

	Moderator	Effect	SE	95% confidence intervals	
				LLCI	ULCI
Direct effect	ICP → VB	0.45	0.12	0.20	0.69
Indirect effect	High RA	0.22	0.07	0.10	0.40
	Low RA	0.42	0.09	0.24	0.61
	Difference	−0.20	0.10	−0.48	−0.07
	Index	−0.13	0.07	−0.32	−0.04

*N* = 259; reported unstandardized effect estimates, related standard errors (SE), and 95% confidence intervals.

## 5 Conclusion

Drawing on the broaden-and-build theory of positive emotions and attribution theory, this study examined the effect of inclusive climate perception on China’s new generation employees’ voice behavior, as well as the mediating (i.e., workplace friendship), moderating (i.e., relational attribution), and moderated mediation processes in the relationship. Inclusive climate perception provides a critical positive emotion facilitator, as proved by its influence on employees’ positive emotion mechanism (i.e., employees build deep workplace friendships through inclusive climate perception, leading to more voice behavior). This study also demonstrated that our findings are contingent upon the relational attribution of the new generation employees. In other words, the positive relationship between inclusive climate perception, workplace friendship, and voice behavior was demonstrated to be accentuated by a low level of relational attribution. These findings carry significant theoretical and practical implications for the domain of inclusion and voice.

### 5.1 Theoretical implications

First, this study emphasized the critical mediating effect of workplace friendship between inclusive climate perception and voice behavior among China’s new generation employees. Under the background of workforce diversity, the positive outcomes (e.g., voice behavior) of inclusive climate have promoted extensive discussion among researchers (Shore et al., 2011). Existing studies have explored the mediation mechanism by satisfying employees’ autonomy, competence, and relatedness needs, increasing psychological safety, and decreasing perceived social loafing (Paolillo et al., 2021; Li et al., 2023). To our knowledge, there is no research that explores the relationship between inclusive climate and voice behavior from the perspective of positive emotions; thus we take the broaden-and-build theory of positive emotions as the theoretical underpinning and find that workplace friendships play a critical mediating role in the relationship between inclusive climate perception and voice behavior among China’s new generation employees. Therefore, this study shows initiative in connecting the literature on inclusive climate, workplace friendships, and voice behavior.

Second, this study confirmed that new generation employees’ differential reactions to inclusive climate perception are related to their relational attribution. Different employees might perceive different extents of inclusive climate. Previous studies used the latent profile analysis method, which is a person-centered tool to identify subgroups of a large heterogeneous group (Bergman and Magnusson, 1997). Using this method, Qu et al. (2021, 2022) found that workplace inclusion has three profiles, namely a high inclusion group, a moderate inclusion group, and a low inclusion group in the Chinese organizational context. However, few studies focus on using a variable-centered method to explore the mechanism of the difference in inclusive climate perception according to individual characteristics. Thus, drawing on attribution theory, the current study found that China’s new generation employees use relational attribution to interpret their perception of inclusive climate, which then influences employees’ workplace friendships. Our finding suggests that it is the followers’ interpretations of organizational inclusive climate that influence the followers’ attitudes and behaviors.

Finally, this study further explores and reveals the intrinsic mechanism of the relationship between inclusive climate perception and voice behavior by constructing a moderated mediation model. We answered the call of Li et al. (2023) that future research should take personality traits or individual differences as moderators to discuss boundary conditions between inclusive climate perception and voice behaviors. Relational attribution belongs to intrapersonal attribution processes (Burton et al., 2014), and emphasizes that individuals attribute events to their relationships with others (Eberly et al., 2011), thus affecting an individual’s subsequent attitudes and behaviors (Sun et al., 2019). Our study reveals that relational attribution moderates the mediating effect of workplace friendship between inclusive climate perception and China’s new generation employees’ voice behavior, such that the lower the relational attribution, the stronger the effect. This further illustrates that an inclusive climate has a stronger compensatory role when relational attribution is low, which matches Nishii’s (2013) viewpoint about inclusive climate.

### 5.2 Practical implications

Our findings in this study provide a critical way of thinking for organizations to encourage new generation employees to speak up more. As a leading force shaping the social and economic dynamics in China, this generation has diverse needs, values, and concerns (Xiao and Cooke, 2012), and organizational managers should create an inclusive climate in the workplace to facilitate these employees’ voice behavior. Organizations could pay more attention to guaranteeing fairness and impartiality in management practices, such as ensuring that each employee has the right to speak up. Meanwhile, organizations need to emphasize differential integration to leverage the true value of new generation employees’ advice, which encourages all employees to make recommendations for organizational sustainable development.

Another practical implication is that organizations should provide an inclusive climate environment for China’s new generation employees, which aims to establish shared objectives, ascertain mutual values, and help them build close bonds with colleagues, thereby forming deeper workplace friendships (Zhang et al., 2021). In addition, HR managers could recruit young employees who are easier to connect with others, or support training lectures about how to forge a positive relationship with colleagues, and improve the communication platform system, including formal and informal systems (Yin et al., 2018).

The last practical implication concerns new generation employees’ relational attribution in an inclusive climate. Self-centered and individualism are the core generational features among new generation employees (Xu et al., 2023). Organizational managers should pay more attention to the extent of new generation employees’ relational attribution and the increasing need for diversity management to make them truly feel inclusiveness in the workplace, which is a stronger compensatory way to build deep friendships with colleagues, especially when relational attribution is low. Meanwhile, for diverse teams such as cognitively diverse teams, leadership development programs could focus on humility behavior and relational communication (Liu S.M. et al., 2021).

### 5.3 Limitations and future research

Some limitations still exist in this study. First, participants in this study were from a high-tech company in Chongqing, China, engaged in software development, cloud computing, and artificial intelligence sectors. Even though there were no serious common method biases, and discriminant validity and data quality tests met statistical measurement requirements, future research should examine and generalize this conceptual model in different countries, work sectors, and different generations.

Second, we used a multi-wave and multi-source research design to collect the research data, and we also designed a scenario event to measure relational attribution orientation under the inclusive climate context. Although these research methods strongly supported the hypotheses of this study and ensured external validity, internal validity might be limited; thus, future research could use a diary study or scenario-based experimental methodology combined with questionnaire methods to guarantee internal and external validity. Moreover, managers’ demographic information should be controlled in future research. In addition, Jing and Zhou (2017) suggested that future research should pay attention to the “too-much-of-a-good-thing” effect of inclusive climate, which means considering the curvilinear effect between inclusive climate perception and voice behavior in follow-up studies.

Third, we only discussed inclusive climate perception from the employee level, which emphasizes employee diversity, while ignoring diversity at the leader level. Gip et al. (2024) raised that there are distinct differences between diversity at employee level and leader level, and they defined perceived inclusion climate for leader diversity (PICLD) as “the degree to which organizational members believe that organizational policies encourage and foster the acceptance of leaders who are demographically diverse (p. 79).” They also developed PICLD scale and suggested that future research should explore the relationship between PICLD and voice behavior (Gip et al., 2024). Future research could compare the effect size of employee-level and leader-level inclusive climate perception on voice behavior.

In addition, Zhu and Warner (2018) found that it is hard to cope with negative leadership for new generation employees. Leader humility is not only a rare personality trait but is also characterized by positive emotions, admitting personal limitations and mistakes, spotlighting follower strengths, and modeling teachability (Owens and Hekman, 2012), thus potentially endangering supportive leader–follower relationships (Morris et al., 2005). Humble leaders communicate through observable verbal and nonverbal cues, such as gestures, postures, and facial expressions, which can be systematically observed and analyzed (D’Errico and Poggi, 2019; Liu Y.S. et al., 2021). Such communicative signals can substantially shape employees’ perceptions of inclusiveness, the quality of workplace friendship, and ultimately their willingness to engage in voice behavior. Prior work has shown the importance of tracking humility through multimodal, behavioral indicators (D’Errico and Poggi, 2019). In this sense, incorporating an observational and micro-analytic perspective on communicative behavior could further strengthen the explanatory power of the model.

## Data Availability

The original contributions presented in the study are included in the article/supplementary material, further inquiries can be directed to the corresponding author.
